# Prevalence of Stroke and Diagnostic Performance of Emergency MRI in Acute Isolated Dizziness

**DOI:** 10.1002/acn3.70195

**Published:** 2025-09-12

**Authors:** Xiao Hu, Sijia Liu, Xiaosan Wu, Sunhong Yan, Jie Shen, Lei Zhu, Xueyun Liu, Zijie Wang, Chu Chen, Tiannan Yang, Chuanqin Fang, Qi Li

**Affiliations:** ^1^ Department of Neurology The Second Affiliated Hospital of Anhui Medical University Hefei China

**Keywords:** computed tomography, dizziness, magnetic resonance imaging, stroke, vertigo

## Abstract

**Objective:**

Stroke is frequently misdiagnosed in patients presenting with acute isolated dizziness; the optimal imaging modality for this population remains debated. This study aimed to determine the prevalence of stroke among patients with isolated dizziness and to assess the diagnostic accuracy of magnetic resonance imaging (MRI) and computed tomography (CT) for stroke detection.

**Methods:**

Consecutive patients presenting to the neuroemergency department with acute dizziness between April and December 2024 were enrolled. Isolated dizziness, defined as dizziness or vertigo without accompanying neurological deficits, was identified. Clinical characteristics were compared between patients with and without stroke. The diagnostic performance of MRI and CT for detecting stroke in patients with isolated dizziness was evaluated.

**Results:**

Among 251 patients with acute dizziness, 129 (51.4%) exhibited isolated dizziness. Of these, 121 (93.7%) underwent emergency MRI. Acute ischemic stroke was diagnosed in 33 out of 129 patients (25.6%) and hemorrhagic stroke in 3 patients (2.3%) with isolated dizziness. Clinical characteristics were similar between patients with and without stroke. The sensitivity and specificity of MRI for detecting acute stroke were 0.939 (95% CI, 0.798–0.993) and 1.000 (95% CI, 0.959–1.000), respectively, whereas CT demonstrated a sensitivity of 0.519 (95% CI, 0.319–0.713) and specificity of 0.795 (95% CI, 0.647–0.902).

**Interpretation:**

Stroke is a frequent but underrecognized cause of isolated dizziness. Clinical features and emergency CT provide limited diagnostic value, whereas MRI offers high accuracy for detecting stroke in this population. Employing MRI as the first‐line imaging modality for patients presenting with dizziness may substantially reduce the risk of misdiagnosis.

## 1 Introduction

Dizziness is one of the most common complaints in the emergency department and is particularly prevalent among patients with acute posterior circulation stroke [1, 2, 3]. However, more than 10% of patients presenting with dizziness are misdiagnosed during their initial evaluation [4], and nearly one‐third of dizziness resulting from cerebrovascular causes may be overlooked following emergency department workups [5]. Patients discharged with a diagnosis of peripheral vertigo face a 50‐fold increased risk of stroke after discharge, underscoring a substantial number of misdiagnoses that may delay optimal treatment [6]. Although the overall reported incidence of missed strokes among patients presenting with dizziness is relatively low, the disproportionately high rate of misdiagnosis for preventable strokes contributes significantly to healthcare burden and worsens patient outcomes [6, 7, 8].

Head imaging and neurological examination are the primary tools used to assess dizziness in acute settings [9]. However, only a small proportion of emergency department physicians report confidence in bedside neurological examinations [10]. Computed tomography (CT) is the most widely used imaging modality in acute settings, but it is insensitive for detecting acute ischemic stroke lesions. Moreover, fewer than half of physicians routinely order head imaging for patients with dizziness, and only 35% of such patients undergo neuroimaging in emergency departments, according to national surveys [10, 11]. Compared with CT, magnetic resonance imaging (MRI) has been demonstrated to be a more accurate technique for diagnosing acute stroke, which may help reduce the risk of misdiagnosis and inappropriate thrombolysis [12, 13]. Despite its advantages, MRI is often underutilized due to cost concerns, potentially leading to missed opportunities for early diagnosis [7, 14].

To address the diagnostic challenges, this study aims to (1) determine the prevalence of acute stroke among patients presenting with isolated dizziness, (2) compare the clinical profiles of stroke and non‐stroke patients within this population, and (3) evaluate the diagnostic performance of MRI and CT in detecting stroke in this cohort.

## 2 Methods

### 2.1 Study Population

We retrospectively analyzed our prospective cohort of all patients admitted to the neuroemergency department between April and December 2024. Patients presenting with acute dizziness within 72 h of symptom onset were included. The study was approved by the Institutional Review Board of the Second Affiliated Hospital of Anhui Medical University. At our institution, patients presenting to the emergency department with neurological symptoms are prioritized for referral to a neurologist following initial screening by nurses. The manuscript conforms to the Strengthening the Reporting of Observational Studies in Epidemiology (STROBE) guidelines. Artificial intelligence was not used in the generation of this manuscript.

### 2.2 Data Collection

Demographic information (age, sex), medical history (hypertension, diabetes, previous stroke, coronary artery disease, and atrial fibrillation), medication (antihypertensives, hypoglycemics, antiplatelet and anticoagulant), systolic and diastolic blood pressure on admission were recorded. Isolated dizziness was defined as the presence of dizziness, vertigo, or imbalance in the absence of impaired consciousness and focal neurological deficits, including visual field defects, facial asymmetry, sensory loss, speech disturbance, dysphagia, aphasia, diplopia, or unilateral limb weakness [5]. The final clinical diagnosis was based on discharge records, incorporating clinical features and findings from both acute and follow‐up neuroimaging, as assessed by experienced neurologists. To aid in distinguishing true transient ischemic attacks (TIA) from mimics, we applied the widely used ABCD^2^ and ABCD^3^‐I scoring systems. A score of ≥ 4 was considered indicative of moderate to high risk for TIA [15, 16].

### 2.3 Imaging Acquisition and Analyses

Our institution has implemented an MRI‐first paradigm for patients with potential cerebrovascular events since April 2023, establishing MRI as the preferred neuroimaging modality, while non‐contrast CT (NCCT) was previously considered the primary method for head imaging. Under the MRI‐first paradigm, patients were transported to available 1.5T or 3.0T MRI scanners at the emergency department. The institutional MRI protocol includes T1‐weighted, T2‐weighted, fluid‐attenuated inversion recovery (FLAIR), diffusion‐weighted imaging (DWI), apparent diffusion coefficient (ADC) mapping, and susceptibility‐weighted imaging (SWI). CT still serves as an alternative imaging modality for patients with MRI contraindications, those with significant motion artifacts, or individuals who declined MRI. Posterior circulation infarction refers to ischemic lesions affecting the brainstem, cerebellum, thalamus, or occipitoparietal lobe.

### 2.4 Statistical Analyses

Continuous variables were presented as medians with interquartile ranges (IQRs), and categorical variables were expressed as frequencies and percentages. Categorical variables were compared between patients with isolated dizziness and those with dizziness accompanied by other neurological deficits using the Chi‐square test or Fisher's exact test, as appropriate. Continuous variables were analyzed using the Student's *t*‐test or the Mann–Whitney *U* test, depending on the distribution of the data. Clinical characteristics were also compared between patients with isolated dizziness who received a final diagnosis of acute stroke and those without stroke. These analyses were repeated in patients with a final diagnosis of acute stroke or transient ischemic attack (TIA) versus those without either diagnosis, as well as in a subgroup of hospitalized patients.

The diagnostic performance of MRI and CT in detecting acute stroke among patients with isolated dizziness was evaluated using sensitivity, specificity, positive predictive value (PPV), negative predictive value (NPV), overall accuracy, and corresponding 95% confidence intervals (CIs). In patients who underwent both MRI and CT in the neuroemergency setting, comparisons of sensitivity and specificity between the two modalities were performed using the Chi‐square test or Fisher's exact test, as appropriate. All statistical analyses were conducted using SPSS software (IBM SPSS Statistics, Chicago, IL, USA) and R software (version 4.2.0; R Foundation for Statistical Computing, Vienna, Austria).

## 3 Results

**FIGURE 1:**
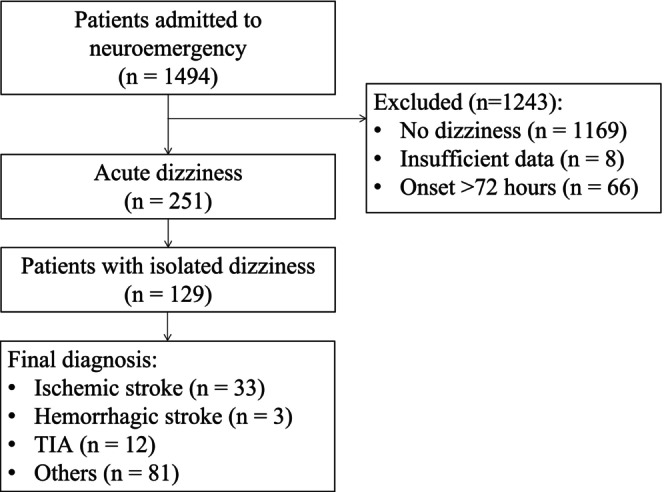
Study flowchart.

Among 1494 patients admitted to the neuroemergency department, 251 (16.8%) presented with acute dizziness within 72 h of symptom onset (median age: 63.0 years; interquartile range [IQR]: 55.0–71.0; 52.2% female; see Table 1 and Figure 1). Of these, 129 patients (51.4%) exhibited isolated dizziness, while the remaining 122 (48.6%) presented with dizziness alongside other neurological deficits. In the emergency room, 231 out of 251 patients (92.0%) underwent MRI, and 149 (59.4%) underwent CT, while 133 (53.0%) patients had both CT and MRI at ED. The median duration of emergency MRI acquisition was 6.0 min (interquartile range: 5.0–7.0 min) in patients with acute dizziness and isolated dizziness (Tables S1 and S2). Compared to those who did not undergo MRI, patients who received ED MRI were generally younger (63 vs. 71 years) and had a shorter time from symptom onset to hospital admission (6.98 vs. 13.58 h; see Table S1).

**TABLE 1:** Clinical characteristics of patients presenting with acute dizziness.

	Total (*n* = 251)	Isolated dizziness (*n* = 129)	Dizziness with neurological deficits (*n* = 122)	*p*
*Clinical characteristics*				
Age, years	63.0 (55.0–71.0)	55.5 (36.0–72.5)	62.0 (54.0–71.0)	0.195
Sex, female	131 (52.2)	64 (49.6)	67 (54.9)	0.400
Previous stroke	71 (28.3)	39 (30.2)	32 (26.2)	0.482
Hypertension	164 (65.3)	77 (59.7)	87 (71.3)	0.053
Diabetes	56 (22.3)	20 (15.5)	36 (29.5)	0.008
Coronary artery disease	19 (7.6)	8 (6.2)	11 (9.0)	0.399
Atrial fibrillation	10 (4.0)	5 (3.9)	5 (4.1)	1.000
Antihypertensives	109 (43.4)	53 (41.1)	56 (45.9)	0.442
Hypoglycemics	44 (17.5)	16 (12.4)	28 (23.0)	0.028
Antiplatelet	40 (15.9)	22 (17.1)	18 (14.8)	0.619
Anticoagulant	7 (2.8)	3 (2.3)	4 (3.3)	0.940
Hospitalization	167 (67.3)	74 (57.4)	93 (78.2)	< 0.001
Systolic BP, mmHg^a^	153.0 (138.0–172.0)	152.0 (136.8–173.0)	155.0 (138.5–172.0)	0.546
Diastolic BP, mmHg^a^	85.0 (78.0–95.0)	85.0 (75.0–96.0)	86.0 (80.0–94.5)	0.301
Onset to admission, hours	7.47 (2.55–24.52)	6.98 (2.67–22.47)	8.48 (2.43–25.36)	0.452
*Imaging technique at ED*				
MRI	231 (92.0)	121 (93.8)	110 (90.2)	0.288
NCCT	149 (59.4)	71 (55.0)	78 (63.9)	0.152
Both MRI and NCCT	133 (53.0)	65 (50.4)	68 (55.7)	0.396
CT angiography	85 (33.9)	32 (24.8)	53 (43.4)	0.002
*Final diagnosis*				
All stroke	111 (44.2)	36 (27.9)	75 (61.5)	< 0.001
Ischemic stroke	99 (39.4)	33 (25.6)	66 (54.1)	< 0.001
ICH	12 (4.8)	3 (2.3)	9 (7.4)	0.061
TIA^b^	22 (8.8)	12 (9.3)	10 (8.2)	0.757
Peripheral vertigo	47 (18.7)	37 (28.7)	10 (8.2)	< 0.001

*Note:* Continuous variables were expressed as median (interquartile range [IQR]), categorical variables as number (percentage).

Abbreviations: BP, blood pressure; CT, computed tomography; ED, emergency department; ICH, intracerebral hemorrhage; MRI, magnetic resonance imaging; NCCT, non‐contrast computed tomography; TIA, transient ischemic attack.

^a^ Blood pressure was not available in 4 patients.

^b^ TIA was diagnosed based on ABCD2 or ABCD3/I score of 4 or more.

### 3.1 Comparison of Patients With Isolated Dizziness Versus Those With Accompanying Neurological Deficits

Table 1 summarizes the demographic and clinical characteristics of patients presenting with isolated dizziness versus those with additional neurological deficits. Of the 251 dizziness cases, 111 (44.2%) were diagnosed with stroke. Notably, acute stroke was less prevalent among patients with isolated dizziness (36/129, 27.9%) compared to those with neurological deficits (61.5%, *p* < 0.001). Conversely, peripheral vertigo was more frequently diagnosed in the isolated dizziness group (28.7% vs. 8.2%). The median onset‐to‐admission time did not significantly differ between groups (isolated dizziness group: 6.98 h [IQR, 2.67–22.47] vs. neurological deficits group: 8.48 h [IQR, 2.43–25.36], *p* = 0.452). Hospitalization rates were also lower in patients with isolated dizziness (57.4% vs. 78.2%, *p* < 0.001; Table 1).

### 3.2 Prevalence of Acute Stroke in Patients With Isolated Dizziness

Among the 129 patients presenting with isolated dizziness, 36 (27.9%) were diagnosed with acute stroke at discharge, including 33 cases (25.6%) of ischemic stroke and 3 cases (2.3%) of intracerebral hemorrhage (Table 1, Figure S1). Of the 33 ischemic stroke cases, 19 (57.6%) were localized to the posterior circulation territory. Infarcts were distributed in the cerebellum in 14 cases (42.4%), basal ganglia in 7 cases (21.2%), cortical regions in 7 cases (21.2%), thalamus in 2 cases (6.1%), and brainstem in 4 cases (12.1%, Table S3).

The characteristics of patients with a final diagnosis of stroke were compared with those of non‐stroke patients with isolated dizziness (Table 2). Patients with stroke tended to be older (68.0 vs. 63.0 years, *p* = 0.116) and had higher systolic blood pressure at admission (158 vs. 149 mmHg, *p* = 0.110). Moreover, no significant differences were observed between the two groups in terms of sex or comorbidities.

**TABLE 2:** Comparison of characteristics of patients with isolated dizziness diagnosed with acute stroke or non‐stroke.

	Acute stroke (*n* = 36)	Non‐stroke (*n* = 93)	*p*
*Clinical characteristics*			
Age, years	68.0 (56.3–74.0)	63.0 (55.0–72.0)	0.116
Sex, female	15 (41.7)	50 (53.8)	0.218
Hypertension	21 (58.3)	56 (60.2)	0.845
Diabetes	6 (16.7)	14 (15.1)	0.820
Coronary artery disease	1 (2.8)	7 (7.5)	0.551
Atrial fibrillation	2 (5.6)	3 (3.2)	0.915
Previous stroke	13 (36.1)	26 (28.0)	0.366
Antiplatelet	7 (19.4)	15 (16.1)	0.653
Anticoagulant	2 (5.6)	1 (1.1)	0.188
Hospitalization	34 (94.4)	40 (43.0)	< 0.001
Systolic BP, mmHg	158.0 (145.0–177.0)	149.0 (134.0–173.0)	0.110
Diastolic BP, mmHg	84.0 (75.0–96.0)	85.0 (77.0–96.0)	0.872
*Imaging technique at ED*			
MRI	33 (91.7)	88 (94.6)	0.828
NCCT	27 (75.0)	44 (47.3)	0.005
Both MRI and NCCT	25 (69.4)	40 (43.0)	0.007
CT angiography	24 (66.7)	8 (8.6)	< 0.001

*Note:* Continuous variables were expressed as median (interquartile range [IQR]), categorical variables as number (percentage).

Abbreviations: BP, blood pressure; CT, computed tomography; ED, emergency department; MRI, magnetic resonance imaging; NCCT, non‐contrast computed tomography.

For sensitivity analysis, we additionally included 12 patients considered at moderate to high risk of TIA under the category of cerebrovascular disease (Table S4). Among patients with acute stroke or TIA, older age (68.0 vs. 61.0 years, *p* = 0.006) and higher systolic blood pressure (158 vs. 146 mmHg, *p* = 0.016) were observed compared with those without stroke or TIA. A marginally higher systolic blood pressure was also noted in patients with stroke or TIA (159.5 [IQR, 147.3–177.0] mmHg vs. 146.5 [IQR, 130.8–169.5] mmHg, *p* = 0.056). Similar trends were observed among patients who were subsequently hospitalized in the stroke unit (Table S5).

### 3.3 Diagnostic Yield of MRI vs. CT

**FIGURE 2:**
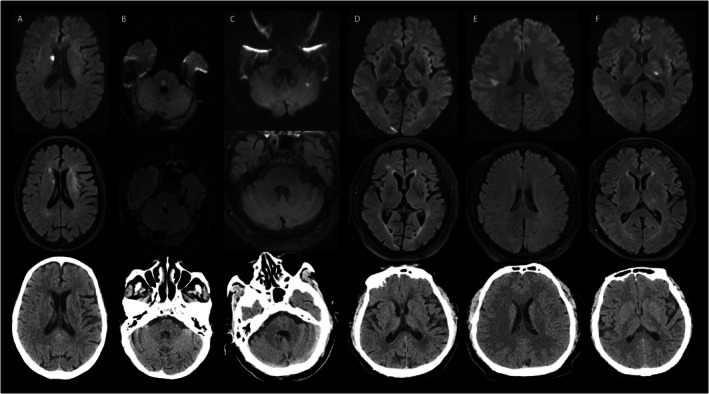
Distribution of ischemic stroke lesions in patients with isolated dizziness across different brain regions. This figure presents diffusion‐weighted imaging, fluid‐attenuated inversion recovery imaging, and computed tomography images of selected patients with isolated dizziness who underwent neuroemergency imaging. Panels A‐F correspond to individual patients.

**FIGURE 3:**
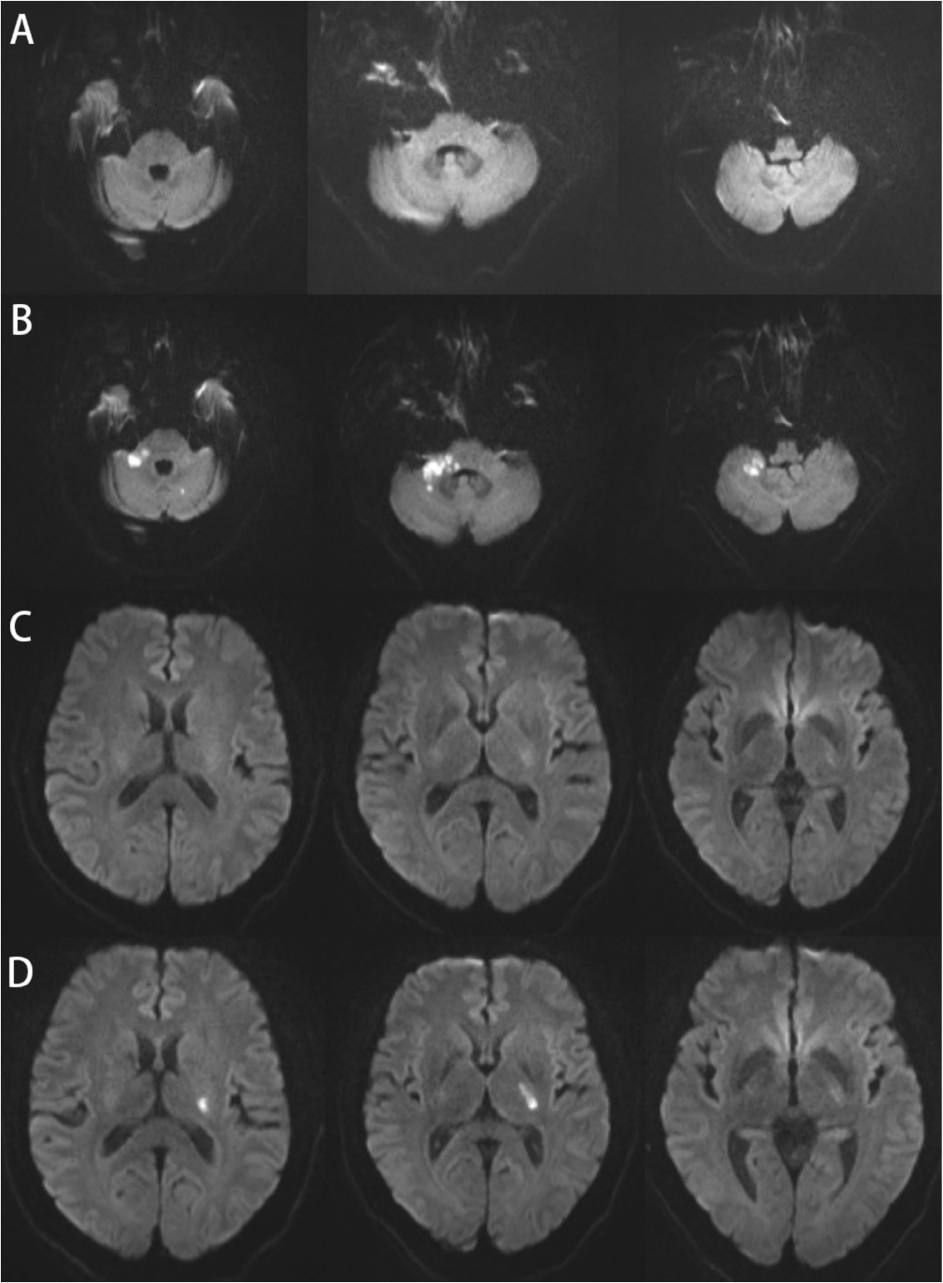
Patients with delayed DWI lesions on follow‐up MRI. (A, B) A patient presented with acute onset vertigo lasting one day. Initial magnetic resonance imaging (MRI) showed no acute ischemic lesions. Follow‐up MRI on day 7 after symptom onset revealed hyperintensity on diffusion‐weighted imaging (DWI) in the bilateral cerebellum and right cerebellar peduncle, confirming an acute ischemic stroke. (C, D) A patient presented with dizziness lasting 22 h. Initial MRI showed no ischemic lesions. Follow‐up MRI one week after admission demonstrated a DWI lesion in the left basal ganglia.

Among the 129 patients presenting with acute isolated dizziness, 121 underwent MRI in the emergency department. The sensitivity, specificity, PPV, NPV, and accuracy for diagnosing acute stroke were 0.939 (95% CI, 0.798–0.993), 1.000 (95% CI, 0.959–1.000), 1.000 (95% CI, 0.888–1.000), 0.978 (95% CI, 0.921–0.997), and 0.983, respectively. In comparison, the corresponding values for NCCT were 0.519 (95% CI, 0.319–0.713), 0.795 (95% CI, 0.647–0.902), 0.609 (95% CI, 0.385–0.802), 0.729 (95% CI, 0.582–0.847), and 0.690, respectively (Table 3). Similar results were also observed in the hospitalized subgroup (Table S6). In patients with isolated dizziness who underwent both MRI and CT at the emergency department (*n* = 65), the corresponding sensitivity, specificity, PPV, NPV, and overall accuracy were 0.480, 0.775, 0.571, 0.705, and 0.662, respectively (Table S7). Figure 2 presents representative emergency MRI images, demonstrating ischemic lesions in isolated dizziness involved both the anterior and posterior circulations, as well as cortical and subcortical regions. Notably, small infarcts are often overlooked on CT scans in the neuroemergency. Only 2 of the 33 patients demonstrated negative findings on the initial MRI but had DWI lesions on follow‐up MRI, despite the absence of newly developed neurological deficits during hospitalization (Table S8, Figure 3).

**TABLE 3:** Diagnostic accuracy of emergency MRI and CT in identifying acute stroke in patients with isolated dizziness.

	MRI (*n* = 121)	CT (*n* = 71)
Sensitivity (95% CI)	0.939 (0.798–0.993)	0.519 (0.319–0.713)
Specificity (95% CI)	1.000 (0.959–1.000)	0.795 (0.647–0.902)
Positive predictive value (95% CI)	1.000 (0.888–1.000)	0.609 (0.385–0.802)
Negative predictive value (95% CI)	0.978 (0.921–0.997)	0.729 (0.582–0.847)
Accuracy	0.983	0.690

Abbreviations: CI, confidence interval; CT, computed tomography; MRI, magnetic resonance imaging.

## 4 Discussion

In this prospective cohort of patients with acute neurological symptoms, nearly 28% of those presenting with isolated dizziness were diagnosed with acute stroke. Clinical profiles did not differ significantly between patients with and without stroke. Moreover, MRI demonstrated significantly higher sensitivity and specificity than CT in detecting acute stroke among patients presenting with isolated dizziness.

The reported prevalence of acute stroke among patients presenting with acute dizziness varies widely, ranging from 0.7% to 62% across different populations. In a study involving 1666 patients with dizziness, 3.2% were diagnosed with stroke or TIA. Among those with isolated dizziness as the chief complaint, only 9 out of 1297 patients (0.7%) received a discharge diagnosis of stroke or TIA [5]. In contrast, a cohort study utilizing subacute MRI identified stroke in 13% of patients with acute vestibular syndrome, and in 5% of those with episodic vestibular syndrome, predominantly due to posterior circulation ischemia [17]. Additionally, a European cohort study of patients presenting with dizziness to a stroke service reported an MRI‐confirmed stroke prevalence exceeding 60% [18]. These rates are notably higher than those observed in earlier studies that relied solely on diagnostic codes and clinical documentation from medical records. In the present study, 36 patients (27.9%) with isolated dizziness (*n* = 129) were diagnosed with acute stroke. The variability in stroke prevalence among dizzy patients may be attributed to differences in the imaging modalities used for initial diagnosis and variations in study methodologies. In patients presenting with acute dizziness, those with accompanying neurological deficits are generally considered at high risk for stroke or transient ischemic attack, whereas individuals with isolated dizziness are typically regarded as having a lower risk of acute cerebrovascular events. In this study, although the incidence of acute stroke was lower in patients with isolated dizziness compared to those with additional neurological deficits (27.9% vs. 61.5%), it remains sufficiently high to warrant greater clinical attention [19].

Among patients with dizziness, only a few reports typical vertigo; instead, most present with symptoms including lightheadedness or gait instability [20]. Consequently, determining the underlying etiology of dizziness relies heavily on comprehensive physical examination and neuroimaging, as chief complaints often offer limited diagnostic value. Evidence suggests that bedside examination serves as an effective screening tool for identifying high‐risk patients and distinguishing between central and peripheral vertigo [9, 21, 22]. However, the diagnostic utility of physical examination techniques, such as the ‘HINTS’ or ‘HINTS plus’, remains limited due to their strong dependence on examiner expertise. The fast‐paced environment of emergency departments further complicates their consistent application. Furthermore, current evidence regarding neurological examination in the diagnosis of acute isolated dizziness is primarily derived from single‐center studies, which limits the generalizability of the findings [10, 23]. Risk stratification tools, such as the ABCD^2^ score, have also shown utility in identifying patients with dizziness who are at higher risk of stroke [24]. Nevertheless, our study did not reveal statistically significant differences in comorbidities between stroke and non‐stroke patients with acute dizziness, consistent with previous findings [25]. These findings indicate that physical examination and clinical characteristics can provide supportive information for the risk stratification of isolated dizziness, but they do not serve as definitive diagnostic tools. This underscores the critical importance of neuroimaging as the most valuable method for accurately diagnosing stroke in patients presenting with dizziness and for guiding clinical decision‐making.

MRI demonstrates higher sensitivity and specificity than CT in the diagnosis of acute stroke [13]. Reliance on non‐contrast CT or the absence of neuroimaging in emergency settings may increase the risk of missed diagnoses, underscoring the critical role of MRI in the accurate evaluation of acute dizziness. Furthermore, compared with CT angiography and CT perfusion, MRI offers the advantages of reduced radiation exposure and minimized use of contrast agents. MRI also provides superior accuracy in detecting intracerebral hemorrhages, particularly for small hematomas and in determining the phase of hemorrhagic lesions [26, 27, 28].

In our study, 19 of the 33 patients with ischemic stroke and isolated dizziness had infarcts localized to the posterior circulation, while the remaining patients primarily exhibited lesions in the basal ganglia and cortical regions. These findings suggest that dizziness is a common manifestation of posterior circulation stroke. Consistent with previous literature, dizziness is also prevalent in hemispheric strokes and should be considered a potential presenting symptom even in the absence of classic signs of posterior circulation involvement [29, 30]. Posterior circulation stroke accounts for approximately one‐fifth of all ischemic strokes [31], with 12% to 25% of affected patients presenting with isolated vestibular symptoms [32, 33]. The performance of CT in detecting posterior circulation stroke is poor, even when combined with CT perfusion [34]. In clinical practice, non‐contrast head CT is often the first‐line imaging modality for patients presenting with atypical stroke symptoms, such as dizziness, vertigo, or headache. However, in cases of posterior fossa infarcts, the sensitivity of non‐contrast CT is generally below 50%, aligning with our findings [31, 35]. In our study, nearly 94% of patients presenting with non‐specific dizziness underwent MRI in the emergency department with the adoption of an MRI‐first approach. Emergency MRI demonstrated a sensitivity exceeding 90%, enabling the detection of ischemic lesions that would not have been identifiable on CT. Although early MRI‐DWI performed within 72 h of stroke onset may yield false‐negative results [9, 36], as observed in our study where 2 of 33 patients with ischemic stroke who had negative findings on initial MRI later exhibited ischemic lesions on follow‐up imaging despite the absence of new symptoms. The proportion of false‐negative cases observed is consistent with previously reported rates of DWI‐negative stroke [37]. These findings highlight the need for future studies to determine the optimal timing for follow‐up MRI in high‐risk patients presenting with acute dizziness, particularly when initial imaging is inconclusive. Nevertheless, if CT is used as the sole imaging modality, nearly half of patients with underlying stroke presenting with acute isolated dizziness may be misclassified as having peripheral or non‐specific vertigo during initial evaluation, potentially leading to missed opportunities for timely revascularization therapy and secondary prevention.

A previous study demonstrated that the early application of MRI within 48 h is associated with improved functional outcomes at discharge and shorter hospital stays in patients with ischemic stroke [38]. Furthermore, implementing MRI as the initial imaging modality has been shown to be both feasible and clinically beneficial, contributing to a reduction in unnecessary thrombolysis for stroke mimics and improved overall diagnostic accuracy [12, 39]. In our center, adoption of an MRI‐first paradigm was associated with a favorable shift in mRS at three months in stroke patients [40]. These findings collectively support the broader implementation of MRI in the acute evaluation of patients with isolated dizziness, as it may enhance diagnostic precision and improve clinical outcomes. Therefore, in well‐equipped stroke centers, adopting an MRI‐first approach for the evaluation of dizzy patients could significantly reduce the likelihood of missed stroke diagnoses and facilitate prompt management.

Our study has several notable strengths. First, we employed MRI as the primary imaging modality for consecutive patients presenting with acute dizziness. By directly comparing the diagnostic yield of MRI and CT for detecting acute stroke in patients with isolated dizziness, this study offers insights into the utility of MRI in emergency care and highlights the limitations of CT in identifying acute stroke in real‐world clinical practice. Second, our findings demonstrate a substantial prevalence of stroke among patients presenting with isolated dizziness, emphasizing the need for heightened clinical vigilance in this population. This study has several limitations. First, we included patients referred to the neuroemergency department. Although individuals presenting with dizziness or vertigo are typically referred to neuroemergency after initial screening by nursing staff at our institution, some patients may have been inadvertently excluded. Second, while MRI was the primary imaging modality in our study, its availability and accessibility may be limited in certain healthcare settings, particularly in resource‐constrained environments. Future studies should explore the feasibility and cost‐effectiveness of implementing an MRI‐first approach across diverse clinical settings. Third, despite the use of emergency MRI, the potential for early false‐negative results in the diagnosis of early ischemic stroke warrants careful consideration. Lastly, as bedside examination findings were not systematically documented in the emergency department records, further studies are needed to explore the potential benefits of integrating structured bedside maneuvers with MRI to improve the diagnostic accuracy in patients presenting with acute dizziness [41].

In conclusion, patients with stroke‐related dizziness are at high risk of misdiagnosis based on clinical presentation and current standard emergency department evaluations. Emergency MRI significantly improved diagnostic accuracy in the acute evaluation of dizziness. Therefore, we recommend the use of MRI as the first‐line imaging modality for patients presenting with acute dizziness to enhance diagnostic precision and support timely clinical decision‐making.

## Author Contributions

Qi Li and Xiao Hu contributed to the conception and design of the study; Xiao Hu, Sijia Liu, and Xiaosan Wu contributed to drafting the text or preparing the figures; Xiao Hu, Sijia Liu, Xiaosan Wu, Sunhong Yan, Lei Zhu, Xueyun Liu, Zijie Wang, Chu Chen and Tiannan Yang contributed to drafting the text or preparing the figures. All authors approved the final version of the manuscript.

## Conflicts of Interest

The authors declare no conflicts of interest.

## Supporting information


**Figure S1:** Hemorrhagic lesions in patients presenting with acute isolated dizziness. Three of the 129 patients with isolated dizziness were diagnosed with acute intracerebral hemorrhage.
**Table S1:** Comparison of clinical characteristics of patients with acute dizziness with vs. without emergency MRI.
**Table S2:** Comparison of clinical characteristics of patients with acute isolated dizziness with vs. without emergency MRI.
**Table S3:** Characteristics of 33 patients with isolated dizziness diagnosed with ischemic stroke.
**Table S4:** Comparison of clinical characteristics of patients with isolated dizziness diagnosed as acute stroke/TIA, or non‐stroke/TIA at discharge.
**Table S5:** Comparison of clinical characteristics of hospitalized patients with isolated dizziness diagnosed as stroke or non‐stroke at discharge.
**Table S6:** Diagnostic accuracy of emergency MRI and CT in identifying acute stroke in hospitalized patients with isolated dizziness.
**Table S7:** Diagnostic accuracy of emergency CT for acute stroke detection in isolated dizziness, with MRI‐confirmed lesions as the reference standard.
**Table S8:** Clinical characteristics of two patients with delayed DWI lesions on follow‐up MRI.

## Data Availability

The de‐identified data supporting this study's findings will be made available upon reasonable request by the corresponding author.
