# Supplementary material for: Prevalence of Stroke and Diagnostic Performance of Emergency MRI in Acute Isolated Dizziness
Source: Ann Clin Transl Neurol. 2025 Sep 12;12(12):2514–22. doi: 10.1002/acn3.70195 (PMC12698943; doi:10.1002/acn3.70195)
Supplement: Supplementary file 1 — Figure S1: Hemorrhagic lesions in patients presenting with acute isolated dizziness. Three of the 129 patients with isolated dizziness were diagnosed with acute intracerebral hemorrhage. Table S1: Comparison of clinical characteristics of patients with acute dizziness with vs. without emergency MRI. Table S2: Comparison of clinical characteristics of patients with acute isolated dizziness with vs. without emergency MRI. Table S3: Characteristics of 33 patients with isolated dizziness diagnosed with ischemic stroke. Table S4: Comparison of clinical characteristics of patients with isolated dizziness diagnosed as acute stroke/TIA, or non‐stroke/TIA at discharge. Table S5: Comparison of clinical characteristics of hospitalized patients with isolated dizziness diagnosed as stroke or non‐stroke at discharge. Table S6: Diagnostic accuracy of emergency MRI and CT in identifying acute stroke in hospitalized patients with isolated dizziness. Table S7: Diagnostic accuracy of emergency CT for acute stroke detection in isolated dizziness, with MRI‐confirmed lesions as the reference standard. Table S8: Clinical characteristics of two patients with delayed DWI lesions on follow‐up MRI. [file ACN3-12-2514-s001.docx]

**Supplementary Material**

**Prevalence of Stroke and Diagnostic Performance of Emergency MRI in Acute Isolated Dizziness**

**Table of contents**

Table S1. Comparison of Clinical Characteristics of Patients With Acute Dizziness With vs. Without Emergency MRI

Table S2. Comparison of Clinical Characteristics of Patients With Acute Isolated Dizziness With vs. Without Emergency MRI

Table S3. Characteristics of 33 Patients with Isolated Dizziness Diagnosed with Ischemic Stroke

Table S4. Comparison of Clinical Characteristics of Patients with Isolated Dizziness Diagnosed as Acute Stroke/TIA, or Non-Stroke/TIA at Discharge

Table S5. Comparison of Clinical Characteristics of Hospitalized Patients with Isolated Dizziness Diagnosed as Stroke or Non-Stroke at Discharge

Table S6. Diagnostic Accuracy of Emergency MRI and CT in Identifying Acute Stroke in Hospitalized Patients with Isolated Dizziness

Table S7. Diagnostic Accuracy of Emergency CT for Acute Stroke Detection in Isolated Dizziness, With MRI-Confirmed Lesions as the Reference Standard

Table S8. Clinical Characteristics of Two Patients with Delayed DWI Lesions on Follow-Up MRI

Figure S1. Hemorrhagic Lesions in Patients Presenting with Acute Isolated Dizziness

**Table S1. Comparison of Clinical Characteristics of Patients With Acute Dizziness With vs. Without Emergency MRI**

|  | With ED MRI  (n=231) | Without ED MRI  (n=20) | P value |
| --- | --- | --- | --- |
| Age, years | 63.0 (55.0-71.0) | 71.0 (63.8-80.8) | 0.003 |
| Sex, female | 108 (46.8) | 12 (60.0) | 0.255 |
| Previous stroke | 64 (27.7) | 7 (35.0) | 0.487 |
| Hypertension | 151 (65.4) | 13 (65.0) | 0.974 |
| Diabetes | 48 (20.8) | 8 (40.0) | 0.089 |
| Coronary artery disease | 15 (6.5) | 4 (20.0) | 0.080 |
| Atrial fibrillation | 8 (3.5) | 2 (10.0) | 0.185 |
| Antihypertensives | 100 (43.3) | 9 (45.0) | 0.882 |
| Antiplatelet | 36 (15.6) | 4 (20.0) | 0.842 |
| Hospitalization | 153 (67.1) | 14 (70.0) | 0.791 |
| Systolic BP, mmHg | 153.0 (138.0-172.0) | 149.5 (129.3-173.5) | 0.443 |
| Diastolic BP, mmHg | 85.0 (78.0-95.0) | 82.0 (74.5-92.8) | 0.505 |
| Onset to admission, hours | 6.98 (2.42-24.18) | 13.58 (7.53-29.06) | 0.042 |
| NCCT at ED | 133 (57.6) | 16 (80.0) | 0.050 |
| CT Angiography at ED | 80 (34.6) | 5 (25.0) | 0.383 |
| MRI interval, minutes | 6.0 (5.0-7.0) | NA | NA |
| **Final diagnosis** |  |  |  |
| All stroke | 99 (42.9) | 12 (60.0) | 0.139 |
| Ischemic stroke | 90 (39.0) | 9 (45.0) | 0.596 |
| ICH | 9 (3.9) | 3 (15.0) | 0.060 |
| TIA | 22 (9.5) | 0 (0.0) | 0.302 |
| Peripheral vertigo | 44 (19.0) | 3 (15.0) | 0.884 |
| BP, blood pressure; CT, computed tomography; ED, emergency department; ICH, intracerebral hemorrhage; MRI, magnetic resonance imaging; NCCT, non-contrast computed tomography; TIA, transient ischemic attack. | | | |

**Table S2. Comparison of Clinical Characteristics of Patients With Acute Isolated Dizziness With vs. Without Emergency MRI**

|  | With ED MRI  (n=121) | Without ED MRI  (n=8) | P value |
| --- | --- | --- | --- |
| Age, years | 65.0 (55.0-72.0) | 70.0 (60.5-85.3) | 0.173 |
| Sex, female | 62 (51.2) | 2 (25.0) | 0.283 |
| Previous stroke | 36 (29.8) | 3 (37.5) | 0.948 |
| Hypertension | 74 (61.2) | 3 (37.5) | 0.343 |
| Diabetes | 19 (15.7) | 1 (12.5) | 1.000 |
| Atrial fibrillation | 4 (3.3) | 1 (12.5) | 0.278 |
| Antihypertensives | 51 (42.1) | 2 (25.0) | 0.559 |
| Hypoglycemics | 15 (12.4) | 1 (12.5) | 1.000 |
| Antiplatelet | 21 (17.4) | 1 (12.5) | 1.000 |
| Hospitalization | 70 (57.9) | 4 (50.0) | 0.948 |
| Systolic BP, mmHg | 152.0 (136.8-173.8) | 145.0 (137.0-161.3) | 0.401 |
| Diastolic BP, mmHg | 85.0 (76.5-96.0) | 76.5 (74.0-90.0) | 0.282 |
| Onset to admission, hours | 6.48 (2.54-22.47) | 13.58 (7.53-22.47) | 0.241 |
| NCCT at ED | 65 (53.7) | 6 (75.0) | 0.421 |
| CT Angiography at ED | 30 (24.8) | 2 (25.0) | 1.000 |
| MRI interval, minutes | 6.0 (5.0-7.0) | NA | NA |
| **Final diagnosis** | | | |
| All stroke | 33 (27.3) | 3 (37.5) | 0.828 |
| Ischemic stroke | 30 (24.8) | 3 (37.5) | 0.704 |
| ICH | 3 (2.5) | 0 (0.0) | 1.000 |
| TIA | 12 (9.9) | 0 (0.0) | 1.000 |
| Peripheral vertigo | 35 (28.9) | 2 (25.0) | 1.000 |
| BP, blood pressure; CT, computed tomography; ED, emergency department; ICH, intracerebral hemorrhage; MRI, magnetic resonance imaging; NCCT, non-contrast computed tomography; TIA, transient ischemic attack. | | | |

**Table S3. Characteristics of 33 Patients with Isolated Dizziness Diagnosed with Ischemic Stroke**

|  | **IS Patients with Isolated Dizziness (n=33)** |
| --- | --- |
| Infarct location |  |
| Basal ganglia | 7 (21.2) |
| Thalamus | 2 (6.1) |
| Cortex | 7 (21.2) |
| Brainstem | 4 (12.1) |
| Cerebellum | 14 (42.4) |
| Posterior circulation stroke | 19 (57.6) |
| Onset to admission, hours | 21.28 (6.02-39.75) |
| Stenosis of large artery ≥ 50%^a^ | 20 (74.1) |
| Ischemic lesion on MRI^b^ | 28 (93.3) |
| Ischemic lesion on CT^c^ | 11 (45.8) |
| Continuous variables were expressed as median (interquartile range [IQR]), categorical variables as number (percentage).  ^a^ CTA was performed at ED or during hospitalization in 27 patients;  ^b^ MRI was performed in 30 patients at neuroemergency, 2 patients who had negative findings on MRI in emergency department developed DWI lesion on follow-up MRI after hospitalization;  ^c^ Emergency CT was performed in 24 patients.  CT, computed tomography; IS, ischemic stroke; MRI, magnetic resonance imaging. | |

**Table S4. Comparison of Clinical Characteristics of Patients with Isolated Dizziness Diagnosed as Acute Stroke/TIA, or Non-Stroke/TIA at Discharge**

|  | **Acute Stroke/TIA**  **(n=48)** | **Non-Stroke/TIA**  **(n=81)** | ***P* value** |
| --- | --- | --- | --- |
| **Clinical Characteristics** |  |  |  |
| Age, years | 68.0 (61.0-74.0) | 61.0 (52.5-71.5) | 0.006 |
| Sex, female | 21 (43.8) | 44 (54.3) | 0.246 |
| Hypertension | 30 (62.5) | 47 (58.0) | 0.616 |
| Diabetes | 8 (16.7) | 12 (14.8) | 0.779 |
| Coronary artery disease | 3 (6.3) | 5 (6.2) | 1.000 |
| Atrial fibrillation | 2 (4.2) | 3 (3.7) | 1.000 |
| Previous stroke | 17 (35.4) | 22 (27.2) | 0.324 |
| Antihypertensives | 21 (43.8) | 32 (39.5) | 0.636 |
| Hypoglycemics | 6 (12.5) | 10 (12.3) | 1.000 |
| Antiplatelet | 10 (20.8) | 12 (14.8) | 0.380 |
| Anticoagulant | 2 (4.2) | 1 (1.2) | 0.643 |
| Hospitalization | 36 (75.0) | 38 (46.9) | 0.002 |
| Systolic BP, mmHg | 158 (147-177) | 146 (130-173) | 0.016 |
| Diastolic BP, mmHg | 87 (74-96) | 85 (77-94) | 0.814 |
| **Imaging technique at ED** |  |  |  |
| MRI | 45 (93.8) | 76 (93.8) | 1.000 |
| NCCT | 32 (66.7) | 39 (48.1) | 0.041 |
| Both MRI and NCCT | 31 (64.6) | 35 (43.2) | 0.019 |
| CT Angiography | 25 (52.1) | 7 (8.6) | <0.001 |
| Continuous variables were expressed as median (interquartile range [IQR]), categorical variables as number (percentage).  TIA was diagnosed based on ABCD2 or ABCD3/I score of 4 or more.  BP, blood pressure; CT, computed tomography; ED, emergency department; MRI, magnetic resonance imaging; NCCT, non-contrast computed tomography. | | | |

**Table S5. Comparison of Clinical Characteristics of Hospitalized Patients with Isolated Dizziness Diagnosed as Stroke or Non-Stroke at Discharge**

|  | **Acute Stroke**  **(n=34)** | **Non-Stroke**  **(n=40)** | ***P* value** |
| --- | --- | --- | --- |
| **Clinical Characteristics** |  |  |  |
| Age, years | 68.0 (56.0-74.0) | 63.0 (56.3-72.8) | 0.252 |
| Sex, female | 20 (58.8) | 19 (47.5) | 0.331 |
| Hypertension | 21 (61.8) | 25 (62.5) | 0.948 |
| Diabetes | 6 (17.6) | 7 (17.5) | 0.987 |
| Coronary artery disease | 1 (2.9) | 2 (5.0) | 1.000 |
| Atrial fibrillation | 2 (5.9) | 1 (2.5) | 0.886 |
| Previous stroke | 12 (35.3) | 12 (30.0) | 0.628 |
| Antihypertensives | 16 (47.1) | 20 (50.0) | 0.801 |
| Hypoglycemics | 4 (11.8) | 6 (15.0) | 0.949 |
| Antiplatelet | 7 (20.6) | 5 (12.5) | 0.347 |
| Anticoagulant | 2 (5.6) | 0 (0.0) | 0.233 |
| Systolic BP, mmHg | 159.5 (147.3-177.0) | 146.5 (130.8-169.5) | 0.056 |
| Diastolic BP, mmHg | 85.5 (74.8-96.0) | 82.0 (77.0-91.5) | 0.487 |
| **Imaging technique at ED** |  |  |  |
| MRI | 32 (94.1) | 38 (95.0) | 1.000 |
| NCCT | 26 (76.5) | 22 (55.0) | 0.054 |
| Both MRI and CT | 25 (73.5) | 20 (50.0) | 0.039 |
| CT Angiography | 5 (12.5) | 24 (70.6) | <0.001 |
| Continuous variables were expressed as median (interquartile range [IQR]), categorical variables as number (percentage).  BP, blood pressure; CT, computed tomography; ED, emergency department; MRI, magnetic resonance imaging; NCCT, non-contrast computed topography. | | | |

**Table S6. Diagnostic Accuracy of Emergency MRI and CT in Identifying Acute Stroke in Hospitalized Patients with Isolated Dizziness**

|  | **MRI**  **(n=70)** | **CT**  **(n=48)** |
| --- | --- | --- |
| Sensitivity (95% CI) | 0.939 (0.798-0.993) | 0.538 (0.334-0.734) |
| Specificity (95% CI) | 1.000 (0.907-1.000) | 0.909 (0.708-0.989) |
| Positive predictive value (95% CI) | 1.000 (0.888-1.000) | 0.875 (0.617-0.984) |
| Negative predictive value (95% CI) | 0.950 (0.831-0.994) | 0.625 (0.437-0.789) |
| Accuracy | 0.972 | 0.708 |
| CI, confidence interval; CT, computed tomography; MRI, magnetic resonance imaging. | | |

**Table S7. Diagnostic Accuracy of Emergency CT for Acute Stroke Detection in Isolated Dizziness, With MRI-Confirmed Lesions as the Reference Standard**

|  | **Both CT and MRI performed at ED**  **(n=65)** |
| --- | --- |
| Sensitivity (95% CI) | 0.480 (0.278-0.687) |
| Specificity (95% CI) | 0.775 (0.615-0.892) |
| Positive predictive value (95% CI) | 0.571 (0.340-0.782) |
| Negative predictive value (95% CI) | 0.705 (0.548-0.832) |
| Accuracy | 0.662 |
| CI, confidence interval; CT, computed tomography; ED, emergency department; MRI, magnetic resonance imaging. | |

**Table S8. Clinical Characteristics of Two Patients with Delayed DWI Lesions on Follow-Up MRI**

|  | **Patient 1** | **Patient 2** |
| --- | --- | --- |
| Sex | Female | Male |
| Age, years | 82 | 55 |
| Hypertension | 1 | 1 |
| Diabetes | 0 | 0 |
| Coronary artery disease | 1 | 0 |
| Atrial fibrillation | 0 | 0 |
| Previous stroke | 1 | 0 |
| Antihypertensives | 1 | 1 |
| Hypoglycemics | 0 | 0 |
| Antiplatelet | 1 | 0 |
| Anticoagulant | 0 | 0 |
| Current smoking | 0 | 1 |
| Current drinking | 0 | 1 |
| Systolic BP, mmHg | 177 | 187 |
| Diastolic BP, mmHg | 90 | 123 |
| CT at emergency department | 0 | 0 |
| Onset to admission, hours | 22.00 | 22.17 |
| Onset to initial MRI, hours | 22.45 | 23.43 |
| Initial to follow-up MRI, hours | 141.62 | 137.67 |
| BP, blood pressure; CT, computed tomography; MRI, magnetic resonance imaging. | | |


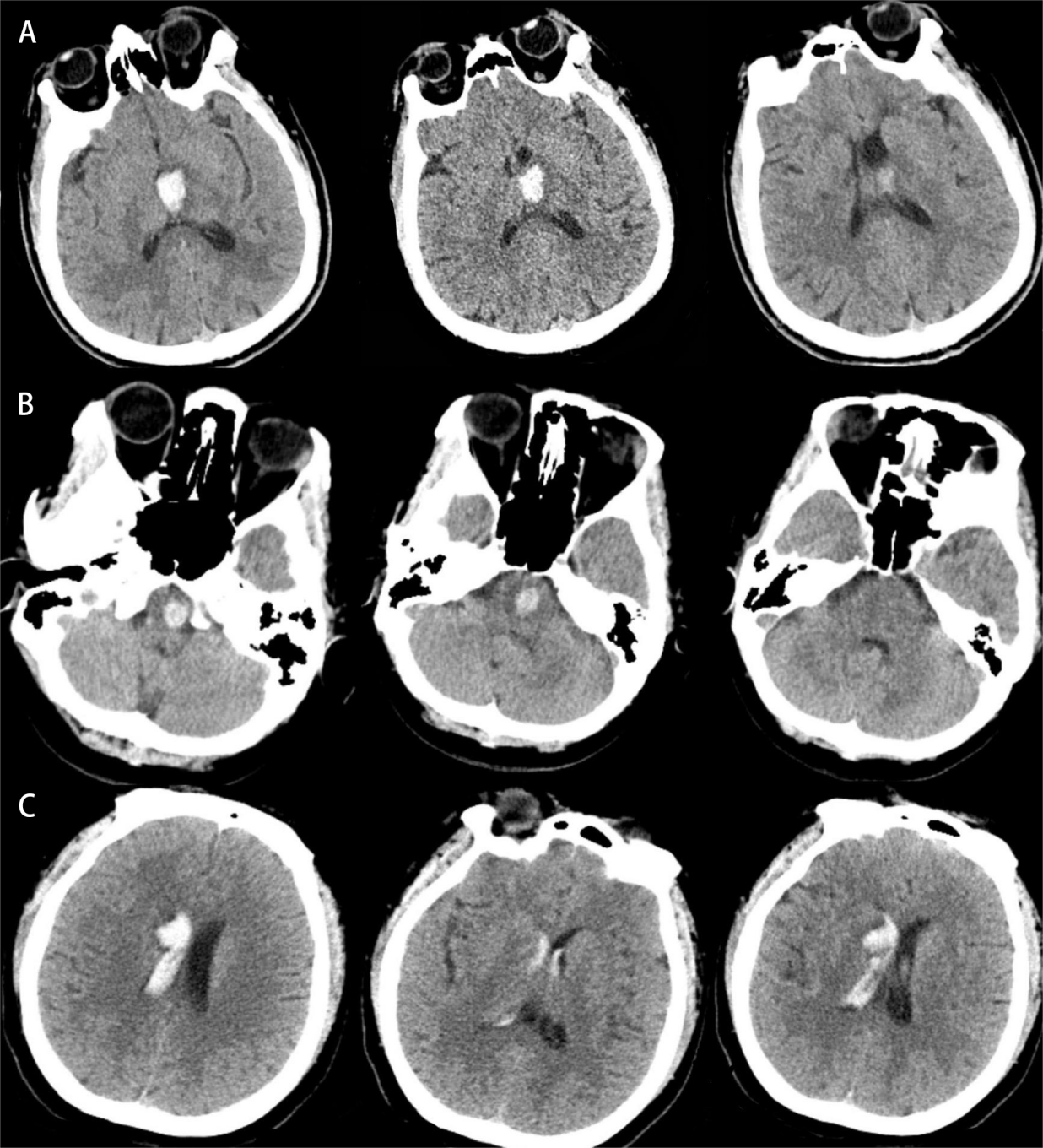


**Figure S1. Hemorrhagic Lesions in Patients Presenting with Acute Isolated Dizziness.** Three of the 129 patients with isolated dizziness were diagnosed with acute intracerebral hemorrhage.
